# Translocation of Methionine Adenosyl Transferase MAT2A and Its Prognostic Relevance for Liver Hepatocellular Carcinoma

**DOI:** 10.3390/ijms24109103

**Published:** 2023-05-22

**Authors:** Pei-Yi Chu, Dev-Aur Chou, Po-Ming Chen, En-Pei Isabel Chiang

**Affiliations:** 1 Department of Post-Baccalaureate Medicine, College of Medicine, National Chung Hsing University, Taichung 402, Taiwan; chu.peiyi@msa.hinet.net; 2 School of Medicine, College of Medicine, Fu Jen Catholic University, New Taipei City 242, Taiwan; 3 Department of Pathology, Show Chwan Memorial Hospital, Changhua 500, Taiwan; 4 National Institute of Cancer Research, National Health Research Institutes, Tainan 704, Taiwan; 5 Department of General Surgery, Changhua Show Chwan Memorial Hospital, Changhua 500, Taiwan; 6 Research Assistant Center, Show Chwan Memorial Hospital, Changhua 500, Taiwan; 7 Department of Food Science and Biotechnology, National Chung Hsing University, Taichung 402, Taiwan; 8 Innovation and Development Center of Sustainable Agriculture (IDCSA), National Chung Hsing University, Taichung 402, Taiwan; 9 Advanced Plant and Food Crop Biotechnology Center (APFCBC), National Chung Hsing University, Taichung 402, Taiwan

**Keywords:** LIHC, GNMT, MAT1A, MAT2A, subcellular localization, prognosis

## Abstract

Methionine adenosyl transferases (MATs) catalyze the synthesis of the biological methyl donor adenosylmethionine (SAM). Dysregulation of MATs has been associated with carcinogenesis in humans. We previously found that downregulation of the *MAT1A* gene enriches the protein-associated translation process and worsens liver hepatocellular carcinoma (LIHC) prognosis. We also discovered that subcellular localization of the MAT2A protein has independently prognostic relevance in breast cancer patients. The present study aimed to examined the clinical relevance of MAT2A translocation in human LIHC. Essential methionine cycle gene expressions in TCGA LIHC datasets were analyzed using Gene Expression Profiling Interactive Analysis 2 (GEPIA2). The protein expression pattern of MAT2A was determined in the tissue array of our own LIHC cohort (n = 261) using immuno-histochemistry, and the prognostic relevance of MAT2A protein’s subcellular localization expression was examined using Kaplan–Meier survival curves. LIHC patients with higher *MAT2A* mRNA expression had a worse survival rate (*p* = 0.0083). MAT2A protein immunoreactivity was observed in both cytoplasm and nucleus fractions in the tissue array. Tumor tissues had elevated MAT2A protein expression in both cytoplasm and nucleus compared to their adjacent normal tissues. A higher cytoplasmic to nuclear MAT2A protein expression ratio (C/N) was found in female LIHC patients compared to that of male patients (*p* = 0.047). Kaplan–Meier survival curves showed that a lower MAT2A C/N correlated with poor overall survival in female LIHC patients (10-year survival rate: 29.2% vs. 68.8%, C/N ≤ 1.0 vs. C/N > 1.0, log-rank *p* = 0.004). Moreover, we found that specificity protein 1 (SP1) may have a potential interaction with nuclear MAT2A protein, using protein–protein interaction; this we found using the GeneMANIA algorithm. We explored the possible protective effects of the estrogen axis in LIHC using the Human Protein Atlas (HPA), and found evidence supporting a possible protective effect of estrogen-related protein ESSRG in LIHC. The localization of SP1 and MAT2 appeared to be inversely associated with ESRRG expression in LIHC. The present study demonstrated the translocation of MAT2A and its prognostic relevance in female LIHC patients. Our findings suggest the potential of estrogen in SP1 regulation and localization of MAT2A, as therapeutic modalities against in female LIHC patients.

## 1. Introduction

Human liver hepatocellular carcinoma (LIHC) is the sixth most frequent carcinoma and the third leading cause of cancer death worldwide, with high prevalence in Eastern Asia and sub-Saharan Africa [1].

**Figure 1:**
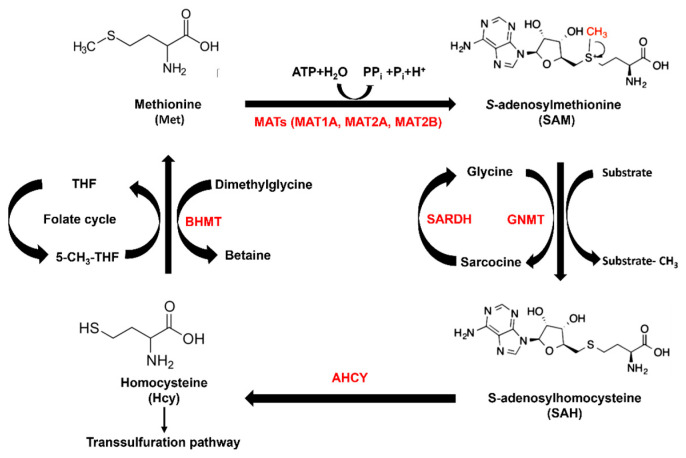
Methionine cycle in human liver. MATs: methionine adenosyl transferases. BMHT: methyl transferase enzyme. GNMT: glycine N-methyltransferase. SARDH: sarcosine dehydrogenase. AHCY: adenosylhomocysteinase.

Methionine is a metabolic necessity of cancer development; thus, methionine availability may hold immense potential for the clinical and therapeutic benefit of LIHC patients. Methionine adenosyl transferases (MATs) catalyze the synthesis of the cellular methyl donor, *S*-adenosylmethionine (SAM), from methionine and ATP (Figure 1). SAM can be used by numerous methyltransferases, including the tetrameric enzyme glycine N-methyltransferase (GNMT), betaine-homocysteine *S*-methyltransferase (BHMT), and many others, with adenosylhomocysteine (SAH) as a reaction product (Figure 1). Three isozymes of MAT are present in mammals [2]. Among the MATs, MAT1 is mainly expressed in the normal adult liver tissues, whereas MAT2 is widely expressed in non-hepatic tissues and in fetal livers [3,4,5,6,7]. During human liver growth, multiple liver diseases, and de-differentiation, MAT1A switches to MAT2A/MAT2B, which will decrease the hepatic SAM level [8]. 

MAT2 enzymes consist of two subunits. MAT2A is the catalytic subunit that can be regulated [9] or stabilized by the other unit of this enzyme, MAT2B [10]. MAT2A can be induced by MYC [11]. Dysregulation of MATs is associated with activation of tumorigenic pathways, chemotherapy resistance, T cell exhaustion, activation of tumor-associated macrophages, and cancer stemness [12]. We have demonstrated that MAT1A expression in liver cancer cells can reduce cell proliferation, invasion of the cell model (through a decrease in the translation-related gene), and eukaryotic translation elongation (EEF1D), and that MAT1A expression can predict a better prognosis in human LIHC [13]. Pérez et al. proposed that oncoprotein P53 and DNA damage-regulated 1 (*PDRG1*) can control the nuclear methylation status through MAT binding, and through its putative collaboration in the progression of hepatic diseases [14]. 

On the other hand, MAT2A is upregulated in proliferative tissues. In partial hepatectomy, MAT2A is upregulated during regeneration [15]. MAT2A has been found in proliferating fetal hepatocytes, and is replaced by MAT1 in adult quiescent hepatocytes [11]. Increased MAT2A expression has also been found in various cancers, including those of the liver [11,13] and breast [16]. Over-expression of MAT2A in LIHC was proposed to be a useful biomarker for predicting and monitoring tumor recurrence, especially early after hepatic resection [17]. 

As the sole methyl donor for methylation of various biomolecules, SAM levels can affect gene expression by changing methylation patterns. Dysregulation of gene promoter methylation has been identified as a potential mechanism for human tumorigenesis, and the association between MAT2A and LIHC is largely due to aberrant methylation. Some translation factors were dynamically methylated in response to the activity of MAT2A [18], indicating that MAT2A is closely related to translation. mTORC1-independent translation was found to be controlled by MAT2A and SAM [18]. A recent study discovered that the mTORC1-c-Myc pathway can rewire methionine metabolism for LIHC progression through suppressing SIRT4-mediated ADP-ribosylation of MAT2A [19]. These studies suggested that MAT2A is required for cancer cell growth and proliferation, and targeting the methionine-MAT2A-SAM axis through MAT2A inhibition could be a novel and promising strategy for cancer therapy [20,21].

MAT2 inhibition reduced polysome formation and decreased the translation efficiency of a fraction of mRNAs. MAT2A was also found to interact with the proteins involved in rRNA processing and ribosome biogenesis; inhibition of MAT2 reduced 18S rRNA processing. Depletion or chemical inhibition of MAT2A reduced protein synthesis in HeLa and Hepa1 cells [18]. Furthermore, nuclear MATα1, the catalytic subunit of MAT1, was found to interact physically and functionally with the *PDRG1* oncogene, resulting in reduced DNA methylation levels. Increased *PDRG1* expression is detected in acute [18] liver injury and hepatoma cells, together with decreased MAT1A expression and nuclear accumulation of MATα1 [14]. Therefore, the link between LIHC and MAT enzymes may go beyond methionine and SAM synthesis. MAT2A protein may provide SAM locally on chromatin, where it interacts with many chromatin-associated proteins with chromatin remodeling and transcription regulation [18].

We have demonstrated that subcellular localization of MAT2A protein is an independent prognostic marker for breast cancer [16]. Breast cancer patients with a higher cytoplasmic to nuclear expression ratio (C/N) of MAT2A protein had lower 5-year survival rates than those with lower C/N ratios. A multivariate Cox regression model analysis further validated the independent prognostic role of MAT2A when patients were grouped by C/N ratio [16].

MAT2A is associated with cancer cell growth and proliferation, and dysregulation of MATs is associated with activation of LIHC. However, it is unclear whether subcellular localization of MAT2A is associated with prognosis of LIHC. Although MAT2A protein was previously found to have a dynamic nuclear localization, the impact of MAT2A nuclear localization and its potential interactions with other proteins have not been investigated in LIHC. The present study aimed to explore the impact of MAT2A expression, and in particular, its subcellular localization, on the prognosis of LIHC. By analyzing the TCGA datasets available within GEPIA2, we discovered that LIHC patients with higher MAT2A had a worse survival rate (*p* = 0.0083) among the methionine cycle enzymes. Additionally, the gene expression profiles of LIHC and adjacent normal liver tissues from TCGA were investigated for MAT2A-related pathways in LIHC occurrence and development. The protein expression pattern of MAT2A in clinical relevance was investigated in the tissue array of our own LIHC cohort (n = 261), using immunohistochemistry. We exposed a novel phenomenon: subcellular localization of MAT2A may affect LIHC metastasis and prognosis, and this is gender-specific. Since the liver is a hormone-sensitive organ that may be regulated by gonadal hormones, the expression patterns of estrogen and related proteins were examined using the Human Protein Atlas (HPA) in LIHC. We also explored other potential mechanisms that might contribute to the clinical observations regarding MAT2A translocation.

## 2. Results

### 2.1. Gene Expression of Methionine Cycle Enzymes and LIHC Overall Survival

**Figure 2:**
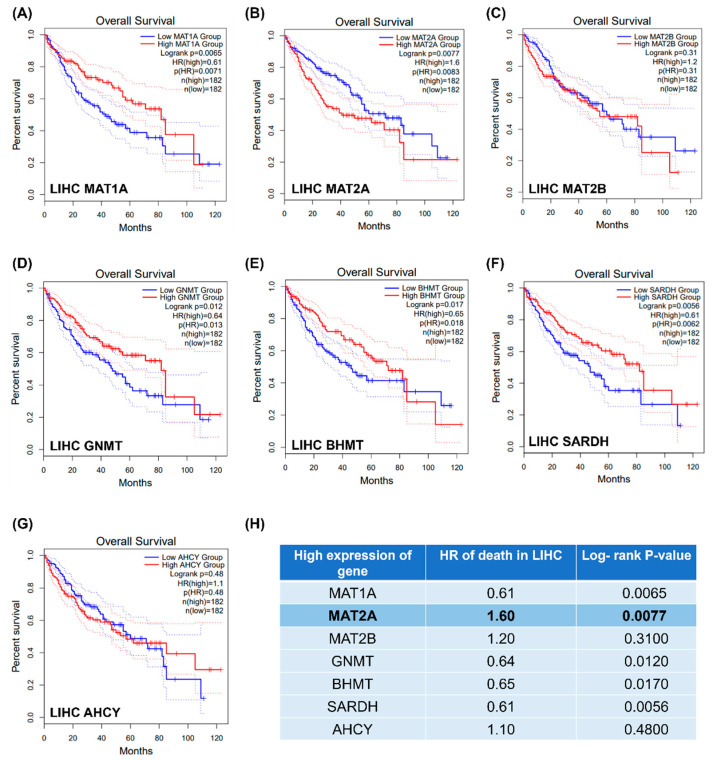
Kaplan–Meier for methionine cycle enzymes in liver hepatocellular carcinoma. (**A**) MAT1A (**B**) MAT2A (**C**) MAT2B (**D**) GNMT (**E**) BHMT (**F**) SARDH (**G**) AHCY (**H**) List of hazard ratios and log-rank *p*-values as high expression of the methionine cycle enzymes. The dotted lines represent the first quartile (Q1) or the lowest quartile (Q4).

The mRNA expressions of the key methionine cycle enzymes, including *MAT1A, MAT2A, MAT2B, GNMT, BHMT, SARDH*, and *AHCY,* were examined using GEPIA web tools (http://gepia2.cancer-pku.cn/#index, accessed on 1 August 2022) (Figure 2A–G).

Among the *MAT* genes, high mRNA expression of *MAT1A* was related to a better LIHC survival (Figure 2A); high mRNA expression of *MAT2A* was associated with poor LIHC survival (Figure 2B), and mRNA expression of *MAT2B* was not related to LIHC survival (Figure 2C). 

LIHC patients with highly expressed *GNMT* had better survival (Figure 2D). Higher expressions of *BHMT* and *SARDH* were also found to be related to a better LIHC survival rate (Figure 2E,F, respectively). mRNA expression of *AHCY* was not related to LIHC survival (Figure 2G). The hazard ratio (HR) of death and log-rank *p*-values of these genes are listed in Figure 2H. The results revealed that those with higher expression of these methionine cycle genes have better LIHC prognoses, apart from in the case of *MAT2A* (Figure 2H); thus, *MAT2A* was selected for further investigation.

### 2.2. MAT2A mRNA Expression in Common Cancers

**Figure 3:**
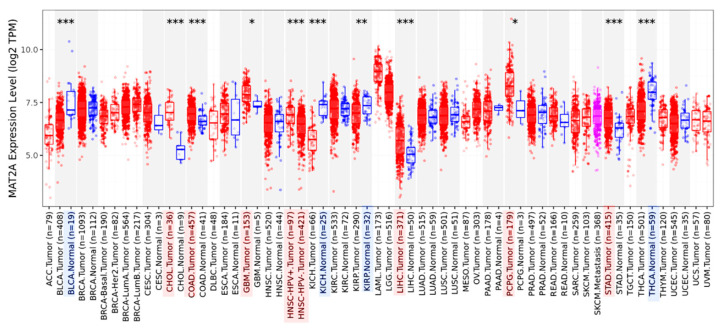
MAT2A mRNA expression in several types of cancers and their normal tissues. * *p* < 0.05, ** *p* < 0.01, *** *p* < 0.001. Red boxes represent tumor tissues and blue boxes represent normal tissues. Light red show that MAT2A expression in tumor tissues was significantly higher. Light blue show that MAT2A expression in normal tissues was significantly higher.

We performed a pan-cancer analysis of *MAT2A* expression using TIMER2.0 (http://timer.cistrome.org/, accessed on 1 October 2022). The analysis revealed that *MAT2A* dysregulation appeared to be present in numerous tumors, including LIHC. *MAT2A* was upregulated in cholangiocarcinoma (CHOL), colon adenocarcinomas (COAD), glioblastoma (GBM), head and neck squamous cell carcinoma (HNSC) with human papillomavirus (HPV) infection, LIHC, pheochromocytoma and paraganglioma (PCPG), and stomach adenocarcinoma (STAD), which were marked in red (Figure 3). On the other hand, *MAT2A* was found to be downregulated in different tumor types, including bladder urothelial carcinoma (BLCA), kidney chromophobe (KICH), kidney renal papillary cell carcinoma (KIRP), and thyroid carcinoma (THCA), which were marked in blue (Figure 3).

### 2.3. Subcellular MAT2A Distributions in Tumor and Adjacent Normal Tissues

**Figure 4:**
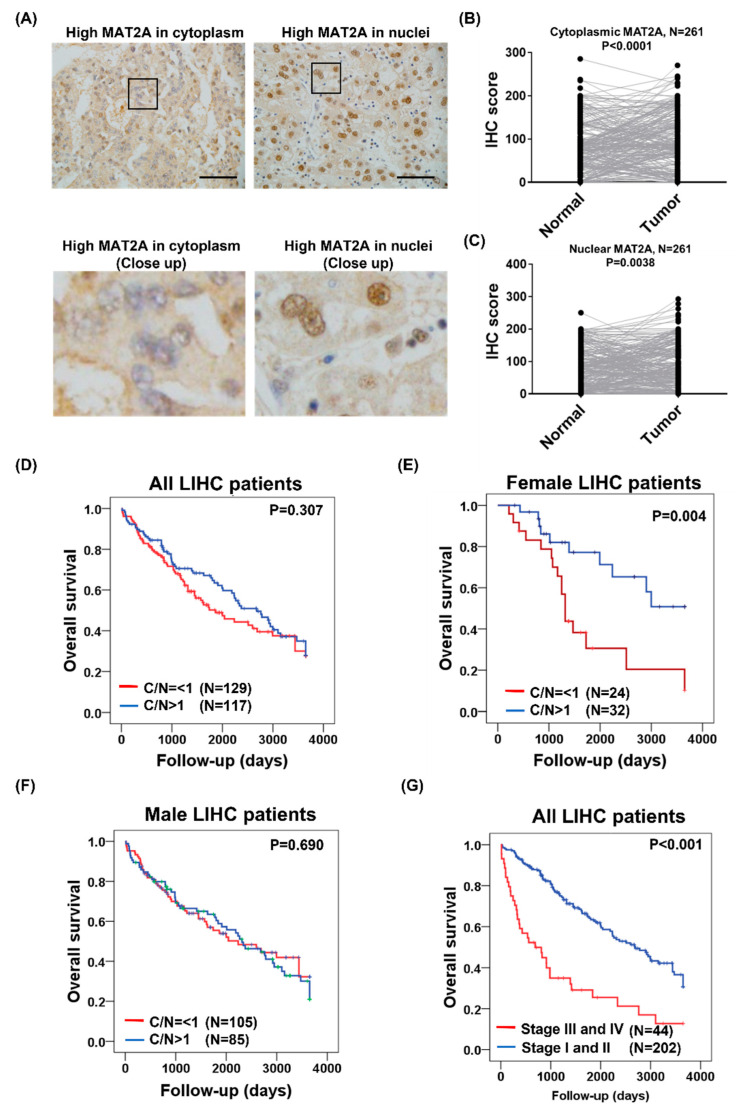
MAT2A immunohistochemical staining in the cytoplasm and nuclei of LIHC. (**A**) Representative graph of immunohistochemistry analysis (400×). LIHC specimens stained the cytoplasmic MAT2A expression in the left panel and the nuclear MAT2A expression in the right panel. (**B**) Cytoplasmic MAT2A was overexpressed in LIHC versus its matched normal liver tissues. (**C**) Nuclear MAT2A is overexpressed in LIHC versus its matched normal liver tissues. (**D**) Overall survival estimated for C/N ratio of MAT2A expression in LIHC, (**E**) in female LIHC, (**F**) in male LIHC, and (**G**) overall survival estimated for LIHC in different stages.

Among the methionine cycle genes examined, *MAT2A* mRNA expression was highly associated with poor survival of LIHC (HR = 1.6, log-rank *p* = 0.0077, Figure 2H). MAT2A protein was found in the nucleus, where it can interact with chromatin-associated proteins [18], and subcellular localization of MAT2A protein was found to be an independently prognostic marker for breast cancer [16]. Therefore, MAT2A was selected for further subcellular immunohistochemistry analyses in our LIHC cohort. A total of 261 independent LIHC tissue sections were stained using the MAT2A antibody. MAT2A protein was found in both the nuclear (N) and cytoplasmic (C) fractions of the tumor and normal tissues. As shown in Figure 1A, the LIHC tumor specimens presented aberrantly high MAT2A expression in both cytoplasm and nuclei. IHC analysis was performed for comparison of MAT2A protein expression between the LIHC tissues and their paired normal liver tissue. MAT2A protein was found to be upregulated in LIHC tissues compared with normal liver tissues in the cytoplasm (*p* < 0.001, Figure 4B), as well as in the nucleus (*p* = 0.0038, Figure 4C).

### 2.4. Subcellular MAT2A Distributions Were Associated with LIHC Survival in Females

The nucleus translocation of MAT2A has been proposed to enable epigenetic histone methylation maintenance during DNA replication in vitro [18]. Furthermore, subcellular localization of MAT2A protein was found to have prognostic application in breast cancer patients [16]. Previous studies on MAT2A translocation and its relevance to breast cancer survival inspired us to explore the prognostic potential and the clinical application of the subcellular localization of MAT2A in our LIHC cohort study. The clinical relevance of a low C/N ratio (C/N ≦ 1) and a high C/N ratio (C/N > 1) of MAT2A were examined. The Kaplan–Meier curve indicated that the C/N ratio of MAT2A expression in the tumorous tissues was not associated with survival rate in all LIHC patients (*p* = 0.307, Figure 4C). However, we discovered that relatively fewer male LIHC patients (46%) had a higher MAT2A C/N compared to that of the females (60.3%, *p* = 0.047, Table 1). The results suggested a possible gender difference in the regulation of MAT2A distribution in LIHC. We then separately examined the prognostic value of MAT2A C/N in both genders. A low C/N of MAT2A expression in the tumorous tissues was found to be significantly associated with poorer survival in females (*p* = 0.004, Figure 4D) but not in males with LIHC. No significant correlation was found between MAT2A C/N in male LIHC (*p* = 0.690, Figure 4E). Stage was expected to be an important factor for LIHC survival (*p* < 0.001, Figure 4F).

### 2.5. Potential Interactions between MAT2A and SP1, WhichIs Negatively Associated with ESSRG

**Figure 5:**
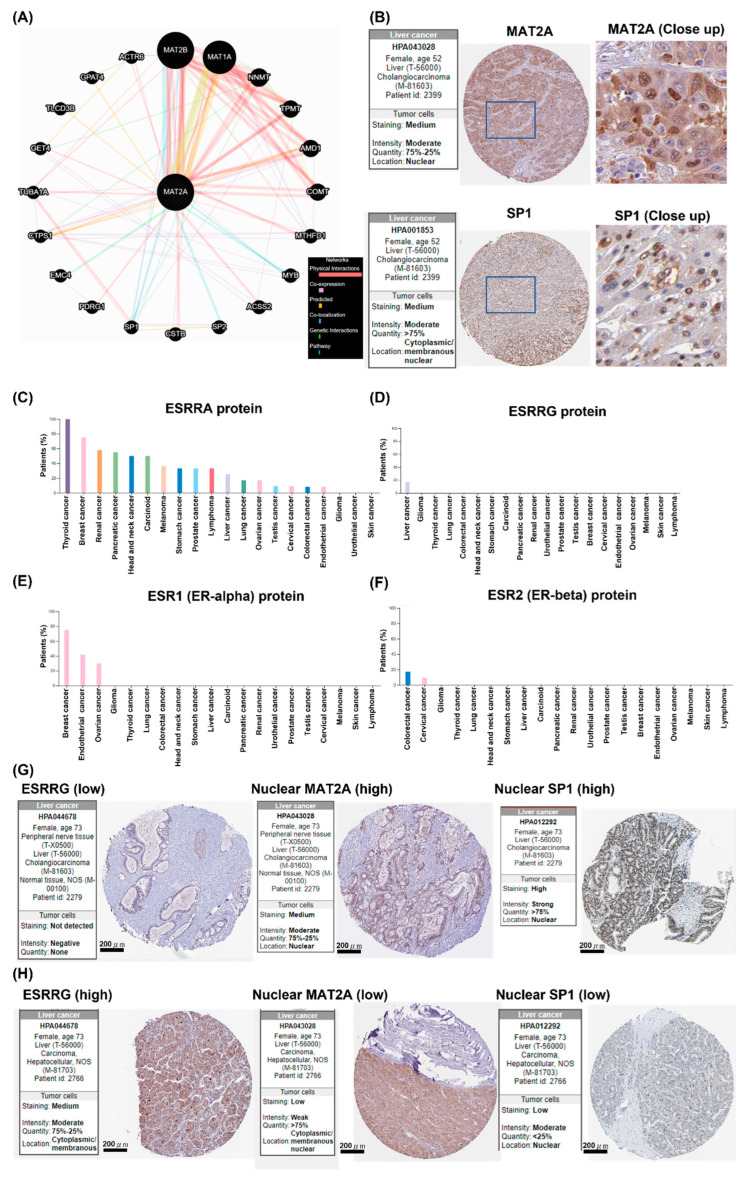
The localization of SP1 and MAT2 is associated with ESRRG in liver cancer. (**A**) The interaction network of MAT2A was explored using the GeneMANIA prediction server. (**B**) The representative images of MAT2A and SP1 IHC are from the same liver cancer patients. (**C**) Most cancers showed weak to moderate cytoplasmic ESRRA positivity. (**D**) A few cases of hepatocellular carcinomas showed moderate cytoplasmic ESRGG immunoreactivity. A fraction of cells in occasional endometrial cancer tissues were moderately ESRGG-stained. The remaining cancer cells were, in general, negative. (**E**) Breast, ovarian and endometrial cancers displayed strong nuclear ESR1 positivity. The remaining cancers were negative. (**F**) Cervical and colorectal cancers showed weak to moderate membranous ESR2 positivity. Most remaining cancer tissues were negative. (**G**) IHC staining of ESRRG, MAT2A, and SP1 molecules with respective antibodies in a patient with cholangiocarcinoma. Scale bars = 200 μm. (**H**) IHC staining of ESRRG, MAT2A, and SP1 molecules with respective antibodies in a patient with LIHC. Scale bars = 200 μm.

The function networks of MAT2A were then predicted using the GeneMANIA algorithm (https://genemania.org/, accessed on 1 October 2022). The correlations between MAT2A and MAT2B, MAT1A, NNMT, TPMT, AMD1, COMT, MTHFD1, MYB, ACSS2, SP2, CSTB, SP1, PDRG1, EMC4, CTPS1, TUBA1A, GET4, TLCD3B, GPAT4, and ACTR6 can be found in physical interactions, co-expression, predicted interactions, colocalization, genetic interactions, and pathways (Figure 5A). We then examined whether MAT2A protein expression was co-localized with the above protein members in the nuclei. We observed a predominant relationship between nuclear co-localization for MAT2A and SP1 protein in LIHC, using HPA. Representative IHC images of MAT2A and SP1 from the same liver cancer patient are shown in Figure 5B.

As a key metabolic organ of the digestive system, the liver is also a hormone-sensitive organ that could be regulated by gonadal hormones. We speculated that the different prognostic values of MAT2A C/N between male and female LIHC patients may be related to sex hormones; thus, we explored whether the estrogen axis may play a role when using MAT2A C/N as a prognostic marker for LIHC. The expression patterns of estrogen and its related proteins, including estrogen-related receptor alpha (ESRRA), ESRRG, ER-alpha (ESR1), and ER-beta (ESR2), were examined in the HPA in different types of cancers. 

Most cancer types showed weak to moderate cytoplasmic positivity of ESRRA proteins compared to cancer of the thyroid and breast (Figure 5C). A few cases of liver cancer (Figure 5D) showed moderate cytoplasmic ESRRG protein immunoreactivity, a fraction of cells in occasional endometrial cancer tissues were moderately stained, and the remaining cancer cells were, in general, negative (https://www.proteinatlas.org/ENSG00000196482-ESRRG/pathology, accessed on 1 October 2022). Strong nuclear ESR1 protein expression was observed in breast, endometrial, and ovarian cancers, but not in other cancers (Figure 5E). Cervical and colorectal cancers showed weak to moderate membranous ESR2 protein expression, and most remaining cancer types were ESR2-negative (Figure 5F).

We also examined MAT2A and SP1 in the HPA online database, and looked for further evidence supporting the possible protective effects of the estrogen-related protein ESSRG, as it was found exclusively in LIHC (Figure 5D). Moderate to high expression of MAT2A and SP1 were present and co-localized in the nuclei in the cancerous tissue from an ESRRG-negative cholangiocarcinoma patient (Figure 5G). On the other hand, nuclear MAT2A and SP1 protein expressions were low in the cancerous tissue from an ESRRG-positive LIHC patient (Figure 5H).

Transcription of the *MAT2A* gene has been found to be up-regulated by Sp1 during proliferation of liver cells [22]; hence, it is possible that the nuclear co-localization of these two proteins may promote LIHC progression and be detrimental to patient survival. The observation that nuclear MAT2A and SP1 protein expressions were low in the cancerous tissue from an ESRRG-positive LIHC patient may partially explain why more female LIHC patients had a higher MAT2A C/N compared to males. It may also explain the survival advantage in female but not in male LIHC patients who have a MAT2a C/N ratio >1. 

**Figure 6:**
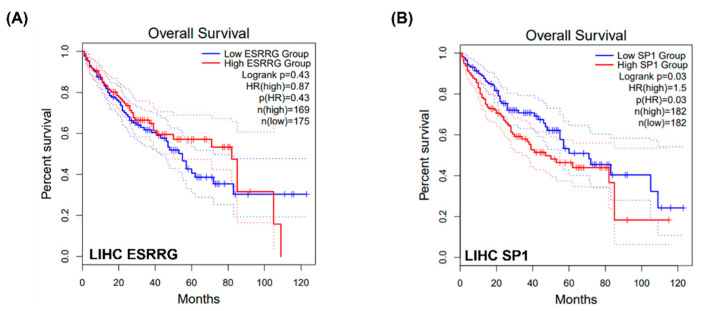
Kaplan–Meier curves for ESRRG and SP1 in LIHC patients. The dotted lines represent the first quartile (Q1) or the lowest quartile (Q4).

**Figure 7:**
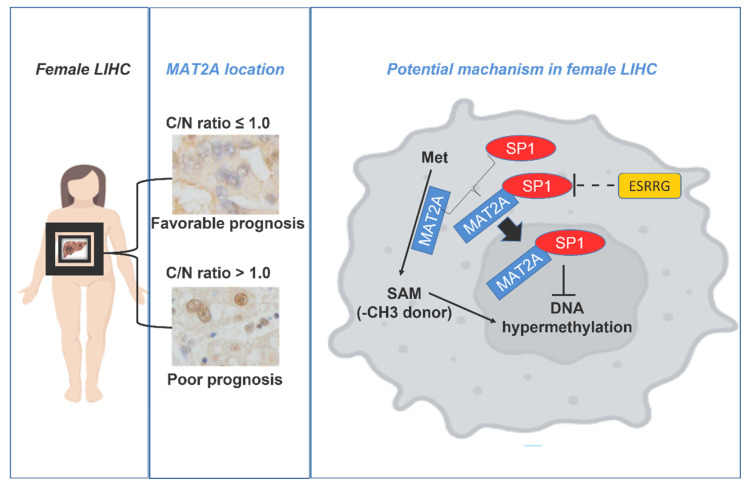
The potential interactions among ESRRG, SP1, and MAT2A in female LIHC.

As low MAT2a C/N was found to be related to poor survival and the nuclear co-localization of these two proteins may promote LIHC progression, the associations of ESRRG (Figure 6A) and SP1 with LIHC survival outcomes were also examined. High SP1 expression exhibited a greater risk when compared to the low SP1 group (Log rank *p* = 0.03, Figure 6B). No statistical significance was found between the binary ESRRG expression and the survival outcome, which might be related to the actual estradiol concentrations in those LIHC patients. The potential interactions among ESRRG, SP1, and MAT2A in female LIHC are shown (Figure 7). 

## 3. Discussion

In this study, we unveiled a novel phenomenon: subcellular localization of MAT2A may affect LIHC metastasis and prognosis, and this is gender-specific. Compared to male LIHC patients, more female patients had a higher cytosol to nucleus ratio of MAT2A protein expression (C/N > 1). Moreover, female patients with higher MAT2A C/N had better survival, and this was found exclusively in female LIHC. When we explored the potential role that gonadal hormones may play in this phenomenon, the potential interactions between MAT2A and SP1 were found to be negatively associated with ESSRG expression in LIHC. This may partially explain why more female LIHC patients had a higher MAT2A C/N compared to males; it may also account for the survival advantage of female LIHC patients with high MAT2a C/N.

Gender difference has been found in LIHC. Up-regulation of tumor MAT1A was independently associated with male gender, and inversely related to tumors over 5 cm [17]. As a key metabolic organ of the digestive system, the liver is also a hormone-sensitive organ that could be regulated by gonadal hormones. Estrogens have been identified as a protective factor for atherosclerotic heart diseases in pre-menopausal women [23]; estrogen can also protect females from LIHC. In mouse models, deletion of *esr1*, the gene that encodes estrogen receptor-alpha, caused the development of 9-fold more tumors than in wild-type mice [24]. Differences in estrogen receptors’ or estrogen receptor-related receptors (ESRRs)’ expression patterns between males and females have been suggested to contribute to the progression of hepatitis C virus (HCV)-related cirrhosis and LIHC [24]. The HPA reported that family members encoded by the *ESRR* genes function as transcriptional activators of *DNA cytosine-5-methyltransferases 1* (*DNMT1*) expression by binding directly to its response elements in *DNMT1* promoters, thereby modulating cell proliferation and estrogen signaling in breast cancer (https://www.proteinatlas.org/ENSG00000196482-ESRRG accessed on 1 October 2022). The expression patterns of estrogen and its related proteins, including estrogen-related receptor ESRRA, ESRRG, ESR1, and ESR2, were therefore examined by HPA in LIHC.

We found that ESRRG protein expression was specifically and exclusively expressed in LIHC. ESRRG has been identified as a candidate tumor suppressor gene in gastric cancer that can inhibit Wnt signaling via the suppression of transcription factor 4 (TCF4)/lymphoid enhancer-binding factor 1 (LEF1), binding to the Cyclin D1 (CCND1) promoter [25]. Hypermethylation of ESRRG promoters contributes primarily to tumor progression and survival prognosis in patients with laryngeal squamous cell carcinoma; ESRRG promoter hypermethylation has also been identified as a diagnostic and prognostic biomarker of laryngeal squamous cell carcinoma [6]. However, mRNA expression of *ESRRG* was not associated with LIHC survival using GEPEA2. Conversely, liver cancer, showing high levels of ESRRG immunoreactivity, was found to be associated with advanced tumor node metastasis, at a late stage and of a high grade, which correlated with poorer overall survival [26]. ESRRG is an orphan receptor (acting as a transcription activator in the absence of a bound ligand) that binds specifically to an estrogen response element and activates reporter genes controlled by estrogen response elements (by similarity). HPA reported that ESRRG is present mainly in cytoplasm, but can also be localized to the nucleoplasm. ESRRG is a favorable prognostic marker in renal cancer. No statistical significance was found between binary ESRRG expression and the survival outcome, which might be related to the impacts of actual estradiol concentrations in different individuals with LIHC. In spite of the gender difference in MAT2A C/N LIHC patients, how ESRRG or other proteins in the estrogen axis participate in the prognosis of MAT2A C/N remains to be further investigated, and more studies are warranted.

We also explored other potential mechanisms that might contribute to the clinical observations regarding MAT2A translocation and LIHC prognosis. The protein PDRG1 has been proposed to control nuclear methylation status through MAT binding and its putative collaboration in the progression of hepatic diseases [14]. PDRG1 was identified as an interacting target for MATα1 (catalytic subunit of MAT1 and MAT3) by yeast two-hybrid and by immunoprecipitation; the protein dynamics of MAT’s regulation by PDRG1 have been reported [14]. PDRG1 was found to interact with MAT2A protein to translocate into the nuclei in cell lines, including CHO (Chinese hamster ovary), COS-7 (monkey kidney), H35 (rat hepatoma), N2a (mouse neuroblastoma) and HEK-293T (human kidney) [14]. The binding of MATs and their putative collaboration with PDRG1 was proposed to control the nuclear methylation status in human hepatoma; therefore, we explored the possible role of PDRG1. However, only a few (urothelial, colorectal, liver, stomach and pancreatic) cancers showed weak to moderate cytoplasmic PDRG1 protein positivity, and remaining malignant tissues were negative in the HPA project (https://www.proteinatlas.org/ENSG00000088356-PDRG1/pathology, accessed on 1 October 2022). Nuclear expression of PDRG1 was not observed in LIHC using HPA, either. Furthermore, no association was observed between overall survival and mRNA expression levels of *PDRG1* in LIHC. These results suggest that PDRG1 only has a minimal role, if any, in the regulation of nuclear MAT2A of LIHC, and PDRG1 is less likely to be accountable for the poor LIHC prognosis of those with low MAT2A C/N.

The low MAT2A in normal liver tissues and high MAT2A expressions in LIHC were consistent with previous studies [3,4,5,6,7,13,17]; yet, the possible impacts of sub-cellular distribution of MAT2A protein on LIHC prognosis remain to be elucidated. Using the GeneMANIA prediction server and HPA, we discovered that SP1 could interact with MAT2A, and the complex may translocate into the nuclei in liver cancer. We also observed a predominant relation of nuclear co-localization for MAT2A and SP1 protein in LIHC. Transcription of the MAT2A gene has been found to be up-regulated by Sp1 during proliferation of liver cells [22]. SP1 plays a vigorous role in promoting carcinogenesis in a variety of tumors, and SP1 up-regulation was reported to predict a poor prognosis for cancer patients [27]. Among the MAT2A interacting proteins, SP1 appeared to co-localize with MAT2A in subcellular fractions; high SP1 expression is also associated with poor LIHC survival. Hence, we speculated that the nuclear co-localization of these two proteins may promote LIHC progression and be detrimental to patient survival. A dysregulated Sp1/miR-130b-3p/HOXA5 axis has been suggested to contribute to tumor angiogenesis and the progression of LIHC [28]. The above studies supported the postulation that SP1 may participate in the poor prognosis of low MAT2A C/N in LIHC. Furthermore, the translocation of MAT2A to the nucleus occurred after G1/S checkpoint that may enable epigenetic histone methylation during DNA replication on cell cycle dynamics in Hela cells [29]. This may serve as another potential mechanism by which subcellular localization of MAT2A modulates LIHC patient prognosis.

The finding of low nuclear MAT2A expression in ESSRG-positive LIHC may account for the close relationship between high MAT2A C/N in female LIHC patients; however, further studies are needed to investigate the mechanism through which a higher MAT2A C/N may predispose female LIHC patients to a better prognosis.

We acknowledge the limitations of present study, in that some of the results were based on bioinformatic analysis, and further in vitro mechanistic studies are needed to determine the proposed interactions among MAT2A, SP1, and ESSRG in LIHC. Nevertheless, data from our LIHC cohort have clearly pointed out the clinical relevance and the gender difference in MAT2A translocation in LIHC. Taken together, we discovered that the subcellular localization of MAT2A protein has independently prognostic relevance in LIHC patients. This is the first study investigating the clinical prognosis potential of MAT2A C/N in human LIHC. The ESSRG expression pattern may partially explain why more female LIHC patients had a higher MAT2A C/N compared to males. It may also explain the survival advantage in female but not male LIHC patients who have a MAT2A C/N > 1. More studies on how MAT2A translocation may affect LIHC are warranted.

## 4. Materials and Methods

### 4.1. Patients

Contralateral primary LIHC and the adjacent normal liver tissues of 261 LIHC patients receiving surgical resection were acquired from Changhua Show Chwan Memorial Hospital. This project was approved by the Ethics Committee of the Institutional Review Board of Show Chwan Memorial Hospital (IRB No. 1100502).

### 4.2. Immunohistochemistry and Scoring

For each patient, representative tissue cores of the LIHC section as well the adjacent normal section were carefully collected and made into a tissue microarray. Immunohistochemistry (IHC) staining was used to evaluate MAT2A protein expression. MAT2A antibody (GTX50027) was purchased from GeneTex, Inc. (Alton Pkwy Irvine, CA, USA). The IHC evaluation and protocol used were previously described in more detail [16].

### 4.3. Correlation Analysis

We used the “Gene_Corr” module of TIMER2.0 [30] (tumor immune estimation re source, version 2) (http://timer.cistrome.org/, accessed on 1 October 2022) to explore the relationships between target genes and the prognosis of LIHC. In the “Gene_DE” module of TIMER2.0, MAT2A was searched, and we observed the expression discrepancy between the tumor and its corresponding normal tissues for the various tumors in the TCGA project.

### 4.4. Protein Analysis

Immunohistochemical (IHC) staining data from the Human Protein Atlas (HPA) (https://www.proteinatlas.org/ accessed on 1 October 2022) were utilized to examine the protein levels of MAT2A, ESRRA, ESRRG, PDGR1, and SP1 in LIHC tumor tissues.

### 4.5. Statistical Analysis

GEPIA2 was performed for survival analyses, as previously described [13,31]. The red blocks denote the higher and blue ones the lower risk. The expression of each gene was extracted from the gene expression profile data, and the samples were divided into high- and low-expression groups according to the median. Log rank *p* values less than 0.05 indicate statistical significance in prognostic analyses. This approach enabled us to screen for the prognostic impact of MAT1A, MAT2A, MAT2B, GNMT, BHMT, SARDH, AHCY, PDRG1, and ESR1 in the LIHC (http://gepia2.cancer-pku.cn/#index, accessed on 1 August 2022). IHC scores of cytoplasmic and nuclear MAT2A were observed in the expression discrepancy between LIHC and its corresponding normal tissues, using a paired sample t test. The Kaplan–Meier plotter is used to assess the correlation between the MAT2A expression ratio of the cytoplasm and nuclei and survival time.

## 5. Conclusions

A lower MAT2A C/N was found to predict a poor survival in female LIHC patients. Our study provides a new indication for MAT2A location in LIHC prognosis. High MAT2A C/N for inhibition of LIHC metastasis and poor prognosis in females may potentially be regulated by ESRRG1 and SP1. Herein, it will be important to consider the role of estrogen in combination with conventional therapies of LIHC, in order to obtain the maximal therapeutic benefits, especially in interventions into metastasis.

## Figures and Tables

**Table 1:** Relationship of clinical parameters with MAT2A protein expression in 261 LIHC.

		MAT2A		
Characteristics	No.	C/N ≦ 1 (N = 132)	C/N > 1 (N = 129)	*p*-Value
Diagnostic age				
<65	134	66	68	0.565
≧65	123	65	58	
Missing	4			
Gender				
Female	63	25	38	0.047
Male	198	107	91	
Stage				
I, II	214	112	102	0.173
III, IV	47	20	27	
10-year follow-up				
Live	119	61	58	0.928
Dead	137	69	68	
Loss of follow-up	15			

Chi-squared test for *p* value.

## Data Availability

The dataset and materials presented in this investigation are available upon request from the corresponding author.

## Abbreviations

MAT	Methionine adenosyl transferases
SAM	Adenosylmethionine
GEPIA	Gene expression profiling interactive analysis
HPA	Human Protein Atlas
LIHC	Liver hepatocellular carcinoma
C/N	Cytoplasmic and nuclear protein expression ratio
BMHT	Methyl transferase enzyme
GNMT	Glycine N-methyltransferase
SARDH	Sarcosine dehydrogenase
AHCY	Adenosylhomocysteinase
SP1	Specificity protein 1
PDRG1	P53 and DNA damage-regulated 1
ESSRG	Estrogen-related receptor G
